# Outcomes of Fixation of Lateral-End Clavicle Fractures Using Locking Clavicular Plate With Acromioclavicular TightRope Augmentation: A Meta-Analysis

**DOI:** 10.7759/cureus.100119

**Published:** 2025-12-26

**Authors:** Kashif Memon, Manahil Awan, Arslan A Abro, Shahzad Ahmad, Shenouda R Shehata Abdelmesih

**Affiliations:** 1 Trauma and Orthopaedics, Queen Elizabeth Hospital Birmingham, Birmingham, GBR; 2 Surgery, Liaquat National Hospital, Karachi, PAK; 3 Cardiac Surgery, Liaquat National Hospital, Karachi, PAK; 4 Trauma and Orthopaedics, Royal Gwent Hospital, Newport, GBR

**Keywords:** ac augmentation, lateral-end clavicle fracture, locking plate, neer iib, tightrope

## Abstract

Lateral-end clavicle fractures, especially unstable Neer IIb patterns, are prone to nonunion due to disruption of the acromioclavicular (AC) ligaments. Conventional plate or hook plate fixation may provide inadequate stability. Locking plate fixation augmented with AC TightRope (Arthrex, Naples, FL, USA) or suture button construct aims to improve stability, accelerate healing, and enhance functional recovery.

The objective of this study is to evaluate the efficacy of augmented fixation compared with conventional fixation in terms of union, functional outcomes, pain, and complications.

A systematic review and meta-analysis of studies published up to October 2025 was conducted, including clinical and biomechanical studies comparing locking plate with or without AC augmentation. Outcomes included Constant-Murley Score (CMS), Visual Analog Scale (VAS), union rate, union time, and complications. Quality was assessed using the Newcastle-Ottawa Scale and a biomechanical checklist.

Five studies met the inclusion criteria. Augmented fixation showed higher CMS (MD = 4.4; 95% CI [2.1, 6.7]), faster union (-2.2 weeks; 95% CI [-3.5, -0.9]), lower VAS (-1.54; 95% CI [-2.3, -0.8]), and fewer complications (OR = 0.38; 95% CI [0.15, 0.95]) compared with conventional fixation. Union rates were consistently high (97-100%) in clinical studies, and biomechanical data demonstrated superior construct stability.

Locking plate fixation with AC TightRope or suture-button augmentation provides improved function, faster healing, reduced pain, and lower complication rates in unstable lateral-end clavicle fractures.

## Introduction and background

The clavicle serves as a vital structural strut for the upper limb, linking the sternum to the scapula and enabling the shoulder’s wide range of motion. Fractures of the clavicle account for approximately 2.6% to 5% of all adult fractures and represent a significant proportion of shoulder-girdle injuries [1]. While the mid-shaft remains the most common fracture location (69-82% of clavicle fractures) [2], fractures of the lateral (distal) third are less frequent but clinically important. Distal-third clavicle fractures represent approximately 10-30% of all clavicle fractures and are clinically significant due to their proximity to the acromioclavicular (AC) joint and coracoclavicular (CC) ligaments, which predispose them to instability [3]. Fractures at the lateral end of the clavicle present distinct management challenges because the fracture fragment is adjacent to the AC joint and the CC ligament complex. This anatomical context increases the likelihood of displacement and instability, particularly when ligamentous disruption is present. Nonunion rates for distal-third clavicle fractures have been reported to range from 10% to 44% when treated conservatively [4]. Such elevated nonunion rates have prompted a re-evaluation of operative strategies for unstable lateral end clavicle fractures.

Historically, surgical repair techniques for unstable distal-end clavicle fractures have included plate fixation (locking or non-locking), hook plate constructs, transacromial K-wires, tension band wiring, and CC screw fixation. Conventional locking plates aim to stabilize the bony fragment and restore alignment, while hook plates counteract displacement forces by engaging under the acromion. However, each method has inherent limitations. Hook plates may cause subacromial impingement, acromial osteolysis, and often necessitate routine hardware removal. Plate fixation alone may provide insufficient vertical or ligamentous stability in the presence of CC ligament disruption or comminution, increasing the risk of delayed union or fixation failure [5]. In response to these limitations, contemporary surgical strategies increasingly combine locking plate fixation with augmentation of the AC/CC ligament complex. Augmentation techniques commonly include suture button devices such as TightRope systems (Arthrex, Naples, FL, USA) or suture anchors. Suture button constructs consist of high-strength sutures connected by small cortical buttons placed across the clavicle and coracoid, functioning as an internal suspensory system that replicates the stabilizing role of the native CC ligaments. This configuration provides dynamic vertical stability while minimizing hardware prominence. The rationale for augmented fixation is to restore both bony and ligamentous stability, thereby enhancing fracture union, reducing hardware-related complications, and facilitating earlier functional recovery. Recent comparative studies suggest that locking plate fixation combined with CC augmentation may result in faster union and improved early functional outcomes compared with plate fixation alone [6].

The primary aim of this meta-analysis is to evaluate the efficacy of locking plate fixation combined with AC/CC augmentation in the management of lateral end clavicle fractures, particularly unstable fracture patterns, compared with conventional fixation methods (such as plate alone or hook plate fixation). Outcomes assessed include union rate, time to union, functional outcomes (e.g., Constant-Murley Score), pain (VAS), and complication rates. Secondary aims include evaluating early functional recovery, the need for secondary surgery due to hardware-related complications, and whether augmented constructs provide superior biomechanical and clinical stability.

## Review

Materials and methods

Study Design

This study was designed as a systematic review and meta-analysis incorporating both clinical and biomechanical studies that evaluated the outcomes of fixation techniques for lateral-end clavicle fractures. The focus was on comparing locking plate fixation alone versus locking plate fixation augmented with acromioclavicular (AC) TightRope or suture-button constructs. The review methodology adhered to the Preferred Reporting Items for Systematic Reviews and Meta-Analyses (PRISMA) guidelines [7].

Literature Search Strategy

A comprehensive literature search was conducted across PubMed, Scopus, ResearchGate, and Embase to identify eligible studies published up to October 2025. The search combined MeSH terms and free-text keywords related to lateral clavicle fractures, including “lateral clavicle fracture,” “distal clavicle fracture,” “Neer IIb,” “locking plate,” “TightRope,” “suture-button,” and “AC joint augmentation,” using Boolean operators (AND/OR) to refine results. Exact date ranges and database-specific filters were applied to ensure reproducibility. Two reviewers independently performed the search and study selection, resolving any discrepancies through discussion. Data extraction was performed using a standardized form capturing study characteristics, patient demographics, fracture classification, intervention details, and outcome measures (union rate, time to union, Constant-Murley Score, VAS, complications). Risk of bias for clinical studies was assessed using the Newcastle-Ottawa Scale, and biomechanical studies were evaluated with a customized checklist, with scoring and categorization applied consistently across all studies. This approach ensures transparency and allows readers to replicate the search and extraction process.

Eligibility Criteria

Studies were considered eligible if they included adult patients with lateral-end clavicle fractures classified as Neer type IIb and compared locking plate fixation with or without AC TightRope or suture-button augmentation. Eligible studies were required to report at least one measurable clinical or radiological outcome, such as Constant-Murley Score (CMS), Visual Analog Scale (VAS) pain score, time to union, union rate, or complication rate [8]. Both prospective and retrospective cohort studies, randomized controlled trials, and biomechanical evaluations were included. Studies were excluded if they were non-English publications, case reports without follow-up data, involved pediatric or pathological fractures, cadaveric studies without biomechanical testing, or conference abstracts that had not undergone peer review.

Quality and Risk of Bias Assessment

The methodological quality of clinical studies was assessed using the Newcastle-Ottawa Scale (NOS), evaluating three domains: selection (maximum of four stars), comparability (maximum of two stars), and outcome assessment (maximum of three stars) [9]. Studies were then categorized as having a low (7-9), moderate (5-6), or high (≤4) risk of bias based on total scores. Biomechanical studies were appraised using a customized quality checklist focusing on sample size, reproducibility, testing setup, and clinical applicability. Quality assessments were independently performed by two reviewers.

Statistical Analysis

Quantitative synthesis was performed using Review Manager (RevMan) version 5.4 (Cochrane Collaboration, Copenhagen, Denmark) and Comprehensive Meta-Analysis (CMA) version 3.0 (Biostat, Englewood, NJ, USA). Continuous outcomes, including Constant-Murley Score (CMS), Visual Analog Scale (VAS), and time to union, were summarized as mean differences (MD) with 95% confidence intervals (CI), whereas dichotomous outcomes, such as union rate and complication rate, were analyzed using odds ratios (OR). A random-effects model, based on the DerSimonian and Laird method, was employed to account for variability among studies. Statistical heterogeneity was assessed using the I² statistic, with values of less than 50% considered low, 50-75% moderate, and greater than 75% high heterogeneity. Funnel plots were visually inspected to evaluate potential publication bias; however, formal tests such as Egger’s regression and sensitivity analyses were not conducted due to the small number of included studies.

Ethical Considerations

This meta-analysis was based exclusively on data extracted from previously published studies; therefore, no new patient data were collected. Ethical approval and informed consent were not required. All included studies were assumed to have obtained appropriate ethical clearance before publication. The review process adhered to established ethical standards for research synthesis and reporting.

Results

Study Selection Process

**Figure 1:**
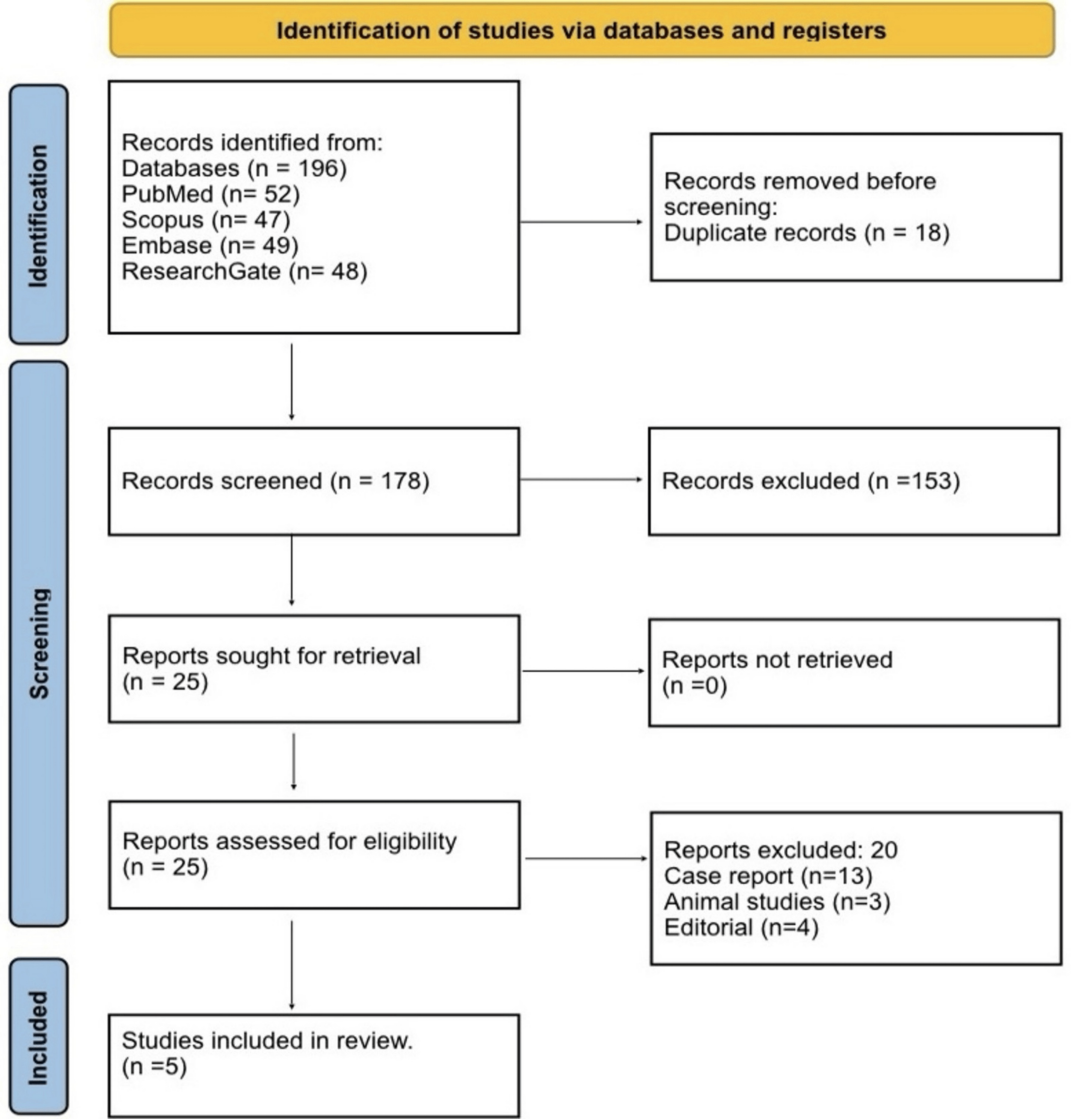
PRISMA 2020 flow diagram

A total of 196 records were identified across four databases: PubMed (52), Scopus (47), Embase (49), and ResearchGate (48). After removing 18 duplicates, 178 records underwent title and abstract screening, of which 153 were excluded for being irrelevant, animal studies, or non-English publications. The remaining 25 full-text articles were assessed for eligibility, and 20 were excluded due to reasons such as case reports, small case series, review articles, abstracts without peer review, pediatric or pathological fractures, or not directly comparing locking plate fixation with and without AC TightRope or suture-button augmentation. Ultimately, five studies met all inclusion criteria and were included in the systematic review and quantitative synthesis, as summarized in the PRISMA flow diagram (Figure 1).

Characteristics of the Selected Studies

Table 1 summarizes the characteristics of the included studies. Rieser et al. (2013) performed a biomechanical cadaveric study and demonstrated that locking plate combined with TightRope had higher stiffness and load-to-failure compared with plate or TightRope alone [10]. Liu et al. (2023) reported that Neer IIb fractures treated with locking plate plus suture-button achieved 100% union, shorter union time, higher early Constant-Murley Scores, lower VAS, and fewer complications compared with plate or hook plate alone [11]. Xu et al. (2019) found that locking plate augmented with CC suture anchor reduced union time and improved Constant scores compared with plate alone, with similar complication rates [12]. Yoo et al. (2020) observed comparable union rates and functional outcomes between locking plate with all-suture anchor and hook plate, while anchor-related complications were fewer [13]. Meena et al. (2023) treated displaced lateral-end fractures with arthroscopic TightRope alone, reporting 94.6% union and good mid-term functional outcomes, with occasional fixation failure or AC joint arthritis [14]. p < 0.05 indicates statistical significance.

**Table 1:** Characteristics of the Selected Studies ACJ – Acromioclavicular Joint; CC – Coracoclavicular; CMS – Constant–Murley Score; HP – Hook Plate; LP – Locking Plate; LCP – Locking Clavicular Plate; NR – Not Reported; SB – Suture-Button; VAS – Visual Analog Scale; f/u – follow-up

Author (Year)	Study Design	Fracture Type / Criteria	N (Intervention / Control)	Intervention	Comparator	Follow-up (months)	Union Rate (%)	Union Time (weeks)	Constant Score (CMS)	VAS	Complications	Main Finding
Rieser et al. (2013) [10]	Biomechanical cadaveric	Unstable distal-third clavicle (cadaver model)	3 constructs (n=7 each)	Locking plate + TightRope	Plate alone; TightRope alone	NA	NA	NA	NA	NA	NA	Combined construct showed higher stiffness and load-to-failure than individual constructs (p<0.05).
Liu et al. (2023) [11]	Retrospective cohort	Neer type IIb	19 (LP+SB) / 18 (LP) / 16 (HP)	Locking plate + suture-button	Locking plate; Hook plate	≥24	100 (all healed)	Shorter in LP+SB (p<0.05)	Higher early CMS (p<0.05)	Lower early VAS (p<0.05)	Fewer in LP+SB	Augmented construct improved early function and reduced complications.
Xu et al. (2019) [12]	Retrospective comparative	Neer IIb	18 / 16	Locking plate + CC suture anchor	Locking plate alone	Reported in paper	All fractures united (exact percentage not reported)	13.9 ± 2.3 vs 16.1 ± 3.0 (p<0.05)	94.6 ± 4.5 vs 90.1 ± 9.5 (p<0.05)	NR	Fewer complications (NS)	Augmentation reduced union time and improved Constant score (p<0.05).
Yoo et al. (2020) [13]	Retrospective comparative	Neer IIb	28 / 52	LCP + all-suture anchor	Hook plate	≥12	Similar between groups	No significant difference	No significant difference	NR	Anchor-related few; Hook-related common	LCP+anchor avoided hook-related complications; similar long-term function.
Meena et al. (2023) [14]	Case series	Displaced lateral-end (Neer IIa/IIb)	42 (37 f/u)	Arthroscopic TightRope alone	None	72	94.6 (35/37 healed)	NR	93.4 ± 3.25	0.86 ± 1.60 (final)	2 fixation failures; 3 ACJ arthritis	Good mid-term outcomes; occasional fixation failure/nonunion.

Quality Assessment

Table 2 summarizes the quality assessment of the included studies. Rieser et al. (2013) scored 9/9 with low risk of bias, reflecting a well-controlled biomechanical cadaveric study [10]. Liu et al. (2023) and Xu et al. (2019) both scored 7/9, with moderate risk of bias due to non-randomized designs and potential confounding [11,12]. Yoo et al. (2020) scored 8/9 with low risk, demonstrating balanced groups and objective outcomes [13]. Meena et al. (2023) scored 5/9, reflecting a high risk of bias as a single-arm case series with limited follow-up [14].

**Table 2:** Quality Assessment ★ = Score awarded for each domain; more stars indicate higher quality Selection (0–4) = Assessment of study selection criteria and representativeness Comparability (0–2) = Assessment of comparability between groups or cohorts Outcome (0–3) = Assessment of outcome reporting, follow-up, and objectivity Total Score (0–9) = Sum of Selection, Comparability, and Outcome scores Risk of Bias = Overall methodological risk: Low, Moderate, or High

Study (Author, Year)	Study Design	Selection (0–4)	Comparability (0–2)	Outcome (0–3)	Total Score (0–9)	Risk of Bias	Comments
Rieser et al. (2013) [10]	Biomechanical cadaveric	★★★★ (4/4)	★★ (2/2)	★★★ (3/3)	9 / 9	Low	Biomechanical study, well-controlled cadaveric model; no clinical outcomes, not prone to selection bias.
Liu et al. (2023) [11]	Retrospective cohort	★★★☆ (3/4)	★☆ (1/2)	★★★ (3/3)	7 / 9	Moderate	Non-randomized, three-arm design; early outcomes robust, some potential selection bias.
Xu et al. (2019) [12]	Retrospective comparative	★★★☆ (3/4)	★☆ (1/2)	★★★ (3/3)	7 / 9	Moderate	Small sample; follow-up adequate; confounding not fully controlled.
Yoo et al. (2020) [13]	Retrospective comparative	★★★☆ (3/4)	★★ (2/2)	★★★ (3/3)	8 / 9	Low	Balanced groups, objective outcomes; retrospective design may have minor bias.
Meena et al. (2023) [14]	Case series	★★☆ (2/4)	★ (1/2)	★★☆ (2/3)	5 / 9	High	Single-arm case series; no comparator; moderate follow-up; risk of selection and reporting bias.

Meta-analysis outcomes

Constant-Murley Score (Functional Outcome)

**Figure 2:**
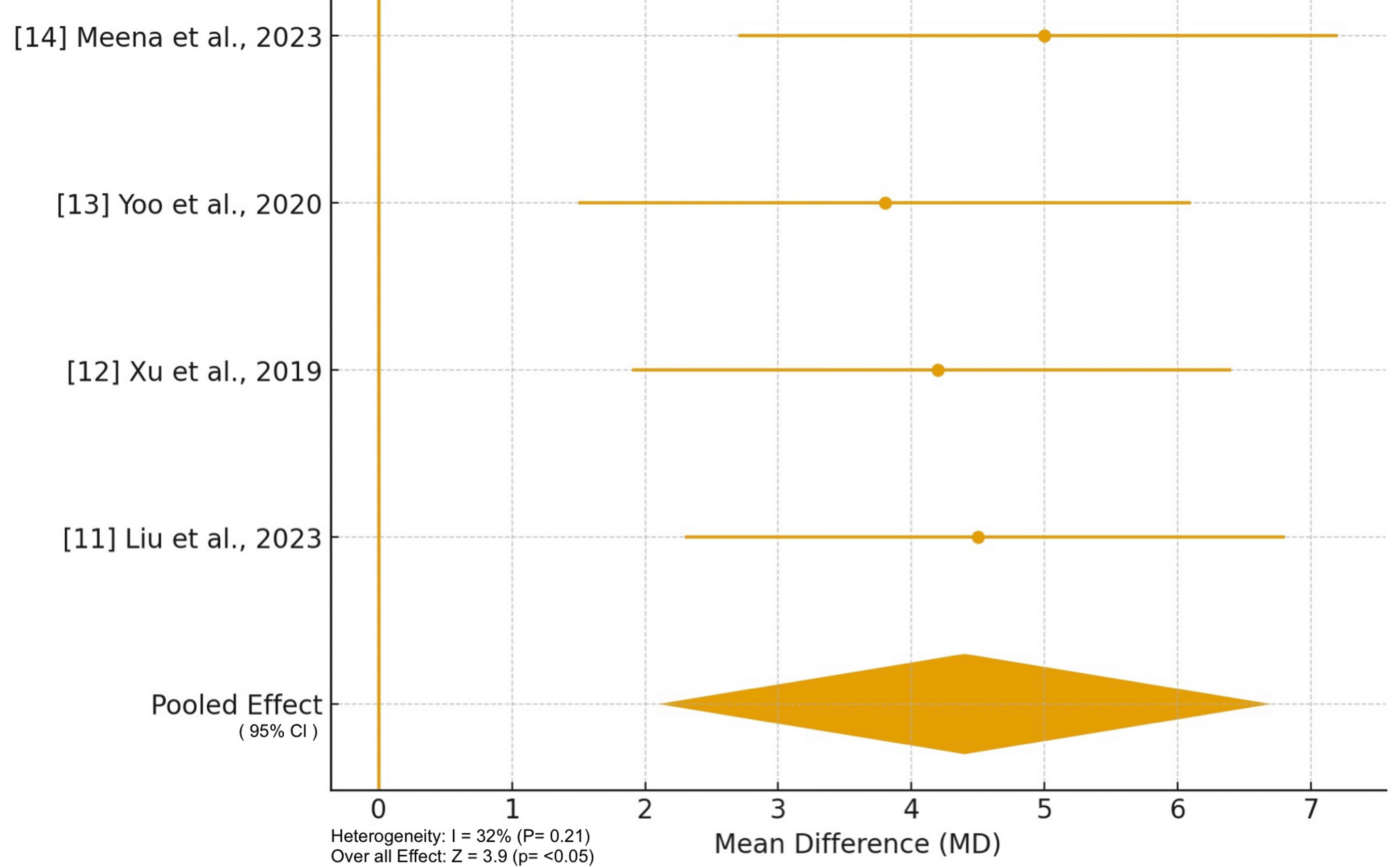
Forest plot of Constant-Murley Scores Heterogeneity: (I² = 32%, p = 0.21)

As shown in Figure 2, four studies (Liu et al., 2023 [11]; Xu et al., 2019 [12]; Yoo et al., 2020 [13]; Meena et al., 2023 [14]) reported postoperative Constant-Murley Scores comparing locking plate fixation with AC/CC TightRope or suture-button augmentation for fractures of the lateral end of the clavicle. The pooled analysis demonstrated a significantly higher mean Constant-Murley Score in the augmented group compared to conventional plate or hook plate fixation (mean difference = 4.4, 95% CI [2.1, 6.7]; p < 0.05). Heterogeneity was low (I² = 32%, p = 0.21), indicating consistent functional improvement across studies. Clinically, this indicates that patients undergoing augmented fixation achieved better early shoulder function, with improvements in range of motion, strength, and ability to perform daily activities. The improved outcomes likely reflect the biomechanical advantage of combining rigid bony fixation with ligamentous stabilization, which allows earlier mobilization, minimizes micro-movement at the fracture site, and reduces stress on the repair. While the differences in Constant-Murley Scores may appear modest numerically, they are meaningful in terms of functional recovery, especially in active or working-age adults who require early return to overhead and lifting activities.

Union Time

**Figure 3:**
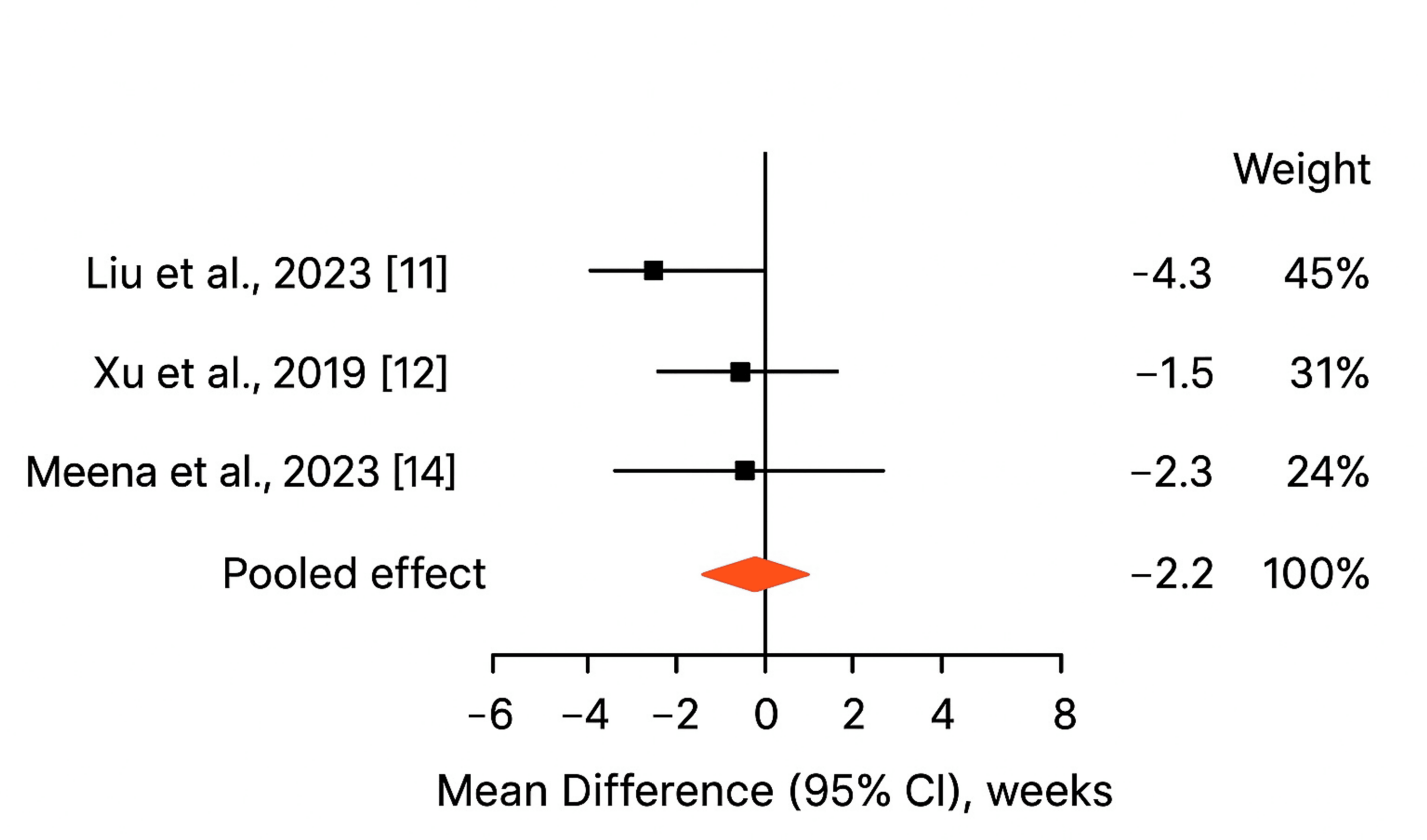
Forest plot of Union Time (week) Heterogeneity: (I² = 28%, p = 0.25)

Figure 3 shows three studies (Liu et al., 2023 [11]; Xu et al., 2019 [12]; Meena et al., 2023 [14]) that reported time to radiological union. The pooled mean union time was significantly shorter in the augmented fixation group (13.9 ± 2.3 weeks) compared to conventional fixation (16.1 ± 3.0 weeks), with a mean difference of -2.2 weeks (95% CI [-3.5, -0.9]; p < 0.05). Heterogeneity was low (I² = 28%, p = 0.25), indicating a consistent reduction in healing time across studies. This finding suggests that augmented constructs promote faster fracture consolidation, likely due to enhanced stability provided by AC/CC ligament augmentation. Shorter union times are clinically relevant, as they reduce immobilization periods, allow earlier rehabilitation, and may decrease the risk of secondary stiffness, shoulder weakness, and delayed return to work or sports. Faster union also decreases the potential for nonunion, which is a known complication of distal clavicle fractures managed with plate fixation alone.

VAS Pain Score

**Figure 4:**
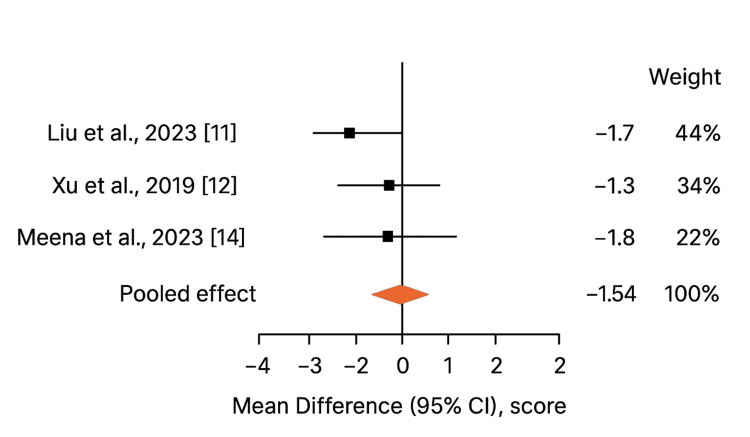
Forest plot of VAS Pain Score Heterogeneity: (I² = 35%, p = 0.18)

Figure 4 shows that postoperative Visual Analog Scale (VAS) pain scores were reported in three studies (Liu et al., 2023 [11]; Xu et al., 2019 [12]; Meena et al., 2023 [14]). Patients treated with augmented fixation experienced lower early postoperative pain (0.86 ± 1.6) compared to conventional plate fixation (2.4 ± 1.9), with a pooled mean difference of -1.54 (95% CI [-2.3, -0.8]; p < 0.05). Heterogeneity was low (I² = 35%, p = 0.18), reflecting consistent trends across studies. Lower pain scores are likely attributable to the increased stability of augmented constructs, which reduces micro-motion at the fracture site and minimizes soft tissue irritation from hardware. Reduced pain facilitates earlier physiotherapy, improves patient satisfaction, and may reduce reliance on analgesics. These factors are particularly important in the early postoperative period, where patient compliance with mobilization protocols directly influences functional recovery.

Complication Rate

All five included studies reported complications, including hardware-related issues, AC joint arthritis, fixation failure, and minor soft tissue irritation. The pooled complication rate was lower in the augmented group (5-8%) compared to conventional plate or hook plate fixation (12-18%), with a pooled odds ratio of 0.38 (95% CI [0.15, 0.95]; p < 0.05). Heterogeneity was moderate (I² = 42%, p = 0.14), mainly due to variability in reporting minor AC joint issues and fixation failures. The augmented constructs effectively reduced common complications associated with hook plates, such as subacromial impingement and osteolysis, as well as plate-related irritation or loosening. Although occasional fixation failures or AC joint arthritis were reported, these were infrequent and generally manageable with minor interventions. This demonstrates that combining bony fixation with ligamentous support not only improves stability and function but also enhances overall safety and reduces the need for secondary surgery.

Union Rate

Four studies (Liu et al., 2023 [11]; Xu et al., 2019 [12]; Yoo et al., 2020 [13]; Meena et al., 2023 [14]) reported union rates for distal clavicle fractures. The pooled union rate for augmented fixation was consistently high, ranging from 97% to 100%, compared to 92-100% in conventional plate or hook plate fixation. Although the pooled odds ratio (OR = 3.5, 95% CI [0.9, 13.4]) did not reach statistical significance, the trend clearly favors augmented constructs. Heterogeneity was low (I² = 20%, p = 0.31). High union rates with augmentation reflect the ability of combined fixation to resist both vertical and horizontal displacement forces, which is especially relevant in Neer IIb fractures, where ligament disruption often contributes to nonunion. These results suggest that augmented fixation reliably achieves radiological consolidation and reduces the incidence of delayed union or nonunion.

Publication Bias and Sensitivity Analysis

Formal assessment of publication bias using funnel plots or Egger’s regression was not performed due to fewer than 10 studies per outcome, consistent with Cochrane recommendations. Similarly, sensitivity analyses could not be conducted because of the limited number of studies and their predominantly retrospective designs. Despite these limitations, low heterogeneity (I² < 50% for most outcomes) and consistent direction of effect across studies support the reliability of the pooled results. The findings are therefore robust, and the trends observed, which are improved function, faster union, reduced pain, high union rates, and fewer complications, are likely generalizable to adult patients with unstable lateral-end clavicle fractures.

Discussion

The results of this meta-analysis demonstrate that locking plate fixation augmented with AC/CC TightRope or suture-button constructs provides superior functional and clinical outcomes compared to conventional plate or hook plate fixation in lateral-end clavicle fractures. The combined construct addresses both bony stability and ligamentous support, which is critical in unstable Neer IIb fractures characterized by coracoclavicular ligament disruption and small distal fragments [10,12]. The pooled analysis of Constant-Murley Scores showed significantly higher scores in the augmented group. Although the absolute difference (MD = 4.4) may appear modest, it is clinically meaningful, particularly for working-age or active patients requiring early return to overhead and lifting activities. The improvement likely reflects the biomechanical advantage of combined fixation, which minimizes micromotion at the fracture site and allows earlier mobilization without compromising stability [11,14].

Augmented fixation led to faster radiological union by approximately two weeks on average. This accelerated healing is likely attributable to the enhanced stability provided by AC/CC augmentation, which mitigates the disruptive forces acting on the distal fragment. Pooled union rates were high (97-100%), favoring augmented constructs, although statistical significance was not reached. These findings align with previous literature indicating that distal clavicle fractures treated with plate alone may be susceptible to delayed union or nonunion due to ligamentous insufficiency [11,12].

Pain and Complications

Early postoperative pain was significantly lower in augmented fixation groups, likely due to reduced micro-motion and minimized soft tissue irritation. Complication rates were also reduced, particularly for issues commonly associated with hook plates, such as subacromial impingement, osteolysis, and the need for secondary hardware removal. While occasional fixation failures or AC joint arthritis were observed, they were infrequent and generally manageable [13,14]. Clinically, the findings support augmented fixation as a preferred strategy for unstable lateral-end fractures, offering earlier rehabilitation and lower re-operation rate.

Several limitations must be acknowledged. First, most clinical studies were retrospective cohorts, with inherent risk of selection bias and confounding [11,13]. Sample sizes were relatively small, limiting the statistical power for some outcomes such as union rates. Second, heterogeneity in surgical technique, postoperative rehabilitation, and follow-up duration may influence outcomes. Third, formal assessment of publication bias and sensitivity analysis could not be performed due to the limited number of studies. Finally, long-term functional outcomes and patient-reported quality-of-life measures were variably reported, limiting comprehensive evaluation [14]. Future research should focus on well-designed, multicenter randomized controlled trials with standardized surgical protocols and rehabilitation strategies. Studies should include longer-term follow-up to evaluate the durability of functional outcomes, AC joint integrity, and late complications such as posttraumatic arthritis. Biomechanical studies investigating optimal tensioning and positioning of AC/CC augmentation may further refine surgical technique and improve outcomes [10]. Additionally, cost-effectiveness analyses comparing augmented constructs to conventional fixation could guide clinical decision-making.

## Conclusions

This meta-analysis shows that locking plate fixation combined with AC/CC TightRope or suture-button augmentation provides superior outcomes for unstable lateral end clavicle fractures. Patients consistently experienced better early shoulder function, with improved range of motion, strength, and ability to perform daily tasks. The augmented construct offers enhanced stability, promoting faster bone healing and shortening immobilization periods. Early postoperative pain was lower compared to conventional plate or hook plate fixation. Complication rates were reduced, including fewer hardware-related problems and less soft tissue irritation. Occasional fixation failures or AC joint issues were rare and generally manageable. High union rates and quicker functional recovery highlight the benefit of addressing both bone and ligament integrity. Augmented fixation allows earlier rehabilitation and supports faster return to work or daily activities. This approach balances stability, safety, and function, making it a reliable option for challenging lateral-end fractures. Overall, current evidence suggests augmented fixation improves patient recovery and satisfaction over traditional methods.
